# Guideline adherence for identification and hydration of high-risk hospital patients for contrast-induced nephropathy

**DOI:** 10.1186/1471-2369-15-2

**Published:** 2014-01-06

**Authors:** Janneke Schilp, Carolien de Blok, Maaike Langelaan, Peter Spreeuwenberg, Cordula Wagner

**Affiliations:** 1 NIVEL, Netherlands Institute for Health Services Research, P.o. box 1568, 3500 BN Utrecht, The Netherlands; 2 Department of Public and Occupation Health, EMGO + Institute for Health and Care Research, VU University Medical Center (VUmc), Van der Boechorststraat 7, 1081 BT Amsterdam, The Netherlands

**Keywords:** Contrast-induced nephropathy, Identification, Hydration, Dutch hospital patient safety program, Guideline adherence, Prevention

## Abstract

**Background:**

Contrast-induced nephropathy (CIN) is a common cause of acute renal failure in hospital patients. To prevent CIN, identification and hydration of high-risk patients is important. Prevention of CIN by hydration of high-risk patients was one of the themes to be implemented in the Dutch Hospital Patient Safety Program. This study investigates to what extent high-risk patients are identified and hydrated before contrast administration. Hospital-related and admission-related factors associated with the hydration of high-risk patients are identified.

**Methods:**

The adherence to the guideline concerning identification and hydration of high-risk patients for CIN was evaluated retrospectively in 4297 patient records between November 2011 and December 2012. A multilevel logistic regression analysis was used to investigate the association between hospital-related and patient-related factors and hydration.

**Results:**

The mean percentage patients with a known estimated Glomerular Filtration Rate before contrast administration was 96.4%. The mean percentage high-risk patients for CIN was 14.6%. The mean percentage high-risk patients hydrated before contrast administration was 68.5% and was constant over time. Differences between individual hospitals explained 19% of the variation in hydration. The estimated Glomerular Filtration Rate value and admission department were statistically significantly associated with the execution of hydration.

**Conclusion:**

The identification of high-risk patients was almost 100%, but the subsequent step in the prevention of CIN is less performed, as only two third of the high-risk patients were hydrated before contrast administration. Large variation between individual hospitals confirmed the difference in hospitals in correctly applying the guideline for preventing CIN.

## Background

Contrast-induced nephropathy (CIN) is the third most common cause of acute renal failure in hospital patients [1]. The incidence of CIN in the general population is 0.6% to 2.3% [2], but when focusing on specific high-risk patients the incidence can increase to more than 40% [3]. CIN is pre-eminently a condition that is potentially suitable for prevention, because it is iatrogenic, its risk factors are well-known and its timing is predictable [4].

There is no universally accepted definition of CIN, but it is usually recognized by an acute deterioration in renal function 2 to 7 days after contrast administration in the absence of an alternative cause of acute renal failure. The most commonly used definition of CIN is an increase in serum creatinine (sCr) of >25% or >44.2 μmol/L (>0.5 mg/dL) within 3 days of intravascular contrast medium administration [5].

CIN has been shown to be associated with an increased risk of prolonged hospital stay, increased risk of nosocomial complications, potential need for dialysis, increased health care costs and mortality [6,7]. Several studies showed effective interventions to decrease the risk for CIN [8,9]. Hydration of high-risk patients for CIN before contrast administration is a universally accepted appropriate and safe measure to prevent CIN [8]. The effect of hydration on the reduction of developing CIN was shown in a few studies [10-15], but only two of these studies included a control group [11,15].

In 2008, the national Dutch Hospital Patient Safety Program (Safety Program) started to improve patient safety and to reduce potentially preventable unintended adverse events in Dutch hospitals [16]. The Safety Program consisted of ten themes regarding patient safety and for each safety theme a module with interventions was developed to support hospitals with the implementation of interventions concerning this theme. Prevention of CIN by hydration of high-risk patients was one of the themes to be implemented. The module for prevention of CIN was based on a national guideline and supported the identification and hydration of high-risk patients for CIN [17,18]. Implementation of guidelines has been proposed to reduce inappropriate care, increase clinical efficiency and better control of health care spending [19,20].

To the best of the authors knowledge, it is unclear to what extent the guideline for prevention of CIN is followed by identification and hydration of patients at high risk for CIN before contrast administration. Furthermore, it is unknown whether factors related to hospitals or admission can be identified whereby hydration is more or less often performed. Only risk factors associated with the development of CIN [21,22] were investigated in previous studies. Factors associated with the subsequent step in the process of preventing CIN were not investigated. Therefore, the aim of the present study was to describe the adherence to the guideline for CIN by evaluating the degree of identification of high-risk patients for CIN and the hydration of high-risk patients during the final year of the Safety Program. Furthermore, in order to gain insight into the factors associated with the hydration of high-risk patients both hospital-related and admission-related factors were investigated.

## Methods

### Study design

The present study was part of an evaluation study of the implementation of the Safety Program in the Netherlands [16]. The evaluation study was a longitudinal retrospective evaluation study, performed during the final year of the Safety Program between November 2011 and December 2012 [23]. A representative sample (n = 38) of hospitals, stratified by area and type of hospital, was drawn from the total sample of 92 Dutch hospitals. The participating hospitals were assigned to three of the ten themes. For each theme, every 4 to 6 weeks a measurement was performed by a trained research assistant during 1 year follow-up, resulting in a total of 10 measurements in each hospital. Data for the present study was collected within 19 hospitals (2 academic, 6 tertiary teaching and 11 general hospitals). Every measurement, a random sample of 20–25 patient records was drawn by the hospital from all adult patients (≥18 y) who have had contrast administration in the month before the measurement.

### Guideline adherence to prevent CIN

The adherence to the guideline for preventing CIN was determined by evaluating to what extent high-risk patients for CIN were identified and hydrated. The identification of high-risk patients for CIN was evaluated by checking in the record if the eGFR (estimated Glomerular Filtration Rate) was known before the contrast administration took place. Following the guideline, the eGFR should be calculated with the MDRD formula, using information of the creatinine concentration, age and sex [18]. The most recent measurement was used to assess the eGFR value and to define whether the patient was at high-risk for CIN, with a maximum durability of the eGFR measurement of 12 months before contrast administration. Following the module for CIN, three categories were defined as high-risk group: 1) eGFR < 45 ml/min/1.73 m^2^; 2) eGFR < 60 ml/min/1.73 m^2^ and diabetes mellitus; 3) eGFR < 60 ml/min/1.73 m^2^ and ≥2 risk factors. Risk factors included in the checklist were: peripheral vascular disease, heart failure, age > 75 year, anemia, symptomatic hypotension, contrast volume > 150 ml, decreased circulating volume, use of diuretics and use of nephrotoxic drugs.

To assess hydration, it was checked in the record whether the patient was hydrated with saline or sodium bicarbonate before contrast administration. Besides hydration, two other prevention measures are mentioned in the guideline, including advising on medication and reducing dose of contrast administration, but these prevention measures were not evaluated in the present study.

### Potential factors associated with hydration

In order to obtain insight into factors associated with hydration of high-risk patients (yes/no) before contrast administration, we assessed both hospital-related characteristics and patient-related characteristics. Hospital-related characteristics were type of hospital (tertiary teaching, academic and general), size of the hospital (number of beds) and admission department. The seven most listed admission departments were allocated: cardiology, urology, internal medicine, surgery, pulmonology and gastroenterology. Different categories were created for those patients listed as day admission whereby no specific department was mentioned, for those listed as not admitted (for example outpatient clinic) or unknown admission, and a category for other less listed departments (for example neurology, gynecology, geriatrics and oncology). Patient-related characteristics were acuteness of admission (yes/no), day admission (yes/no) and eGFR value at admission (continuous).

### Data analysis

The percentage patients with a known eGFR before contrast administration, the percentage high-risk patients, and the percentage high-risk patients who were hydrated were calculated. Descriptive analyses were performed to compare the characteristics of the group at high risk and the group not at high risk for CIN. Chi-square tests were used to test the difference for dichotomous and categorical variables, and t-tests were used to test the difference for continuous variables.

A multilevel logistic analysis was conducted to analyze the trend in hydration of high-risk patients. A two-level multilevel structure was used, whereby the observations were clustered within hospitals. Time was modeled by adding ten indicator variables for the moments (removing the intercept from the model), trends were tested using polynomial contrast (to the 4th order) to study development over time. The intraclass correlation coefficient (ICC) was calculated to indicate the correlation of the observations within the same hospital. An ICC of 20% was seen as moderate [24]. To assess the association between both hospital-related and patient-related factors and hydration separate multilevel logistic regression analyses were performed using hydration as dependent variable and the explanatory factors as independent variables. Categorical independent variables were analyzed by adding separate indicator variables for the categories to the model. In the second analyses the multilevel association models were all corrected for age and sex.

Descriptive analyses were performed using Stata version 12.1 and the multivariate analyses were executed with MlwiN version 2.24.

## Results

### Patient characteristics

A total of 4297 patient records were included in the evaluation study. Mean age was 65.4 (SD 13.9) years and 54% was male. The mean length of hospital stay was 3.6 (SD 10.7) days and 23% of the patients was acutely admitted to the hospital.

The percentage of patients with a known eGFR before contrast administration was 96.4% (n = 4141). The mean eGFR in this sample was 66.6 ml/min/1.73 m^2^. A statistically significant difference in known eGFR (p < 0.001) was found between the hospital types. The percentage patients with a known eGFR was 93.8% in academic hospitals, 95.3% in tertiary teaching hospitals and 97.2% in general hospitals. No statistically significant difference (p = 0.06) was found between the hospital departments, ranging from 94.6% in “other” departments to 97.3% in surgery, day admission and no admission/unknown departments.

Following the criteria of the module, 627 of the 4141 patients (14.6%) were assessed as high-risk patients for CIN (Table 1). Those at high risk were statistically significantly older, less often admitted for one day, more often acutely admitted, had a longer hospital stay, a lower eGFR-value, and more risk factors (mainly high age, use of nephrotoxic drugs/diuretics, heart failure, diabetes mellitus and peripheral vascular disease).

**Table 1:** Characteristics of patients (not) at high risk for contrast-induced nephropathy

**Characteristics**	**Patients at high risk**	**Patients not at high risk**	**P**^**a**^
	**n = 627**	**n = 3514**	
Sex, % male	54.5	54.1	0.831
Age in years, mean (SD)	74.9 (9.5)	64.0 (13.7)	< 0.001
Length of stay in days, mean (SD)	4.7 (8.6)	3.4 (11.1)	0.004
Day admission, %	32.4	39.2	0.005
Acute admission, %	27.7	22.1	0.002
eGFR in ml/min/1.73 m^2^, mean (SD)	44.0 (11.6)	70.6 (17.1)	< 0.001
Risk factors for CIN, %			
0 risk factors	1.1	42.0	< 0.001
1 risk factor	9.7	26.6	
2 risk factors	31.3	15.8	
≥3 risk factors	57.9	15.7	
Diabetes mellitus	31.4	13.3	< 0.001
Peripheral vascular disease	20.4	9.6	< 0.001
Heart failure	39.2	19.9	< 0.001
Age > 75 year	52.3	18.8	< 0.001
Anemia	8.9	2.7	< 0.001
Symptomatic hypotension	1.6	0.5	0.002
Contrast volume > 150 ml	1.6	0.8	0.054
Decreased circulating volume	0.16	0.03	0.169
Use of diuretics	41.3	15.1	< 0.001
Use of nephrotoxic drugs	52.0	30.4	< 0.001

*Abbreviations*: *CIN* Contrast-induced nephropathy.

^a^Tested by chi-square tests (categorical variables) or Student’s t-tests (continuous variables).

### High-risk patients and hydration

Table 2 shows the percentage high-risk patients and percentage hydrated high-risk patients in the hospital admission departments. For the analysis of hydrated high-risk patients, those with a missing value on hydration were excluded (n = 46), resulting in a sample of 581 patients. The mean percentage high-risk patients was 14.6%, and ranged between 7% in case of no admission/unknown to 38% for day admission. The mean percentage hydrated high-risk patients was 68.5%, ranging from 36.9% in case of no admission/unknown to 94.4% at the urology department.

**Table 2:** Percentage high-risk patients and hydration of high-risk patients in hospital admission departments

**Admission department**	**Total**	**High-risk patients**	**Hydration high-risk patients**
	**n**	**n (%)**	**n (%)**^**a**^
Internal medicine	371	89 (24.0)	69 (81.2)
Urology	68	18 (26.5)	17 (94.4)
Cardiology	890	158 (17.8)	92 (63.5)
Surgery	299	59 (19.7)	42 (85.7)
Pulmonology	119	18 (15.1)	12 (80.0)
Gastroenterology	220	23 (10.5)	14 (63.6)
Day admission	261	99 (37.9)	79 (82.3)
No admission/unknown	1647	110 (6.7)	38 (36.9)
Other departments^b^	96	52 (12.3)	35 (72.9)
Total	4297	627 (14.6)	398 (68.5)

^a^Because in 46 high-risk patients information was missing about hydration, the number of high-risk patients used to calculate the percentage hydration differed from the number of high-risk patients in the third column.

^b^Such as neurology, gynecology, geriatrics, intensive care, orthopedics, radiology and oncology.

### Multilevel analyses

**Figure 1:**
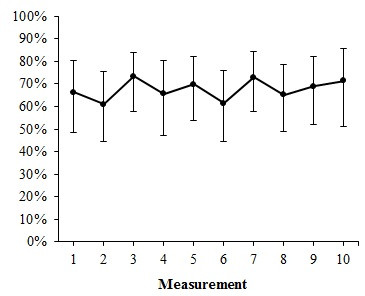
Trend of percentage hydrated high-risk patients (n = 581).

Figure 1 shows the trend over time for hydration of high-risk patients during the final year of the Safety Program. No statistically significant trend (first to the fourth-order multilevel) was found over the study period. The multi-level analysis shows that 19% (ICC = 18,89) of the total variance in hydration was caused by differences between individual hospitals.

All high-risk patients for whom hydration was known were selected for the multilevel analysis for the association between hospital-related factors and patient-related factors and hydration, except those with missing values for age or sex (n = 20), resulting in a sample of 561 patients.

The association between potential explanatory factors and hydration was investigated. The eGFR value was not linearly associated with hydration and was therefore categorized based on quartiles. Hospital type, hospital size, day admission and acute admission were not associated with hydration. There was a negative association between the department no admission/unknown and hydration (Table 3), meaning that high-risk patients not (or unknown) admitted were on average less often hydrated compared to the reference department internal medicine. The ICC was 33.31, meaning that 33% of the total variance in the association between admission departments and hydration was caused by differences between individual hospitals. The estimate remained statistically significant when correcting for age and sex, and the ICC slightly decreased to 32.38.

**Table 3:** Multi-level analysis of the association between admission department and hydration of high-risk patients (n = 561)

	**Model 1**^**a**^	**Model 2**^**b**^
	**Estimate (SE)**	**Estimate (SE)**
**Fixed effects**		
Successful hydration (constant)	1.45 (0.45)	1.46 (0.44)
Admission department		
Internal medicine	*Reference*	*Reference*
Urology	1.01 (1.15)	1.04 (1.16)
Cardiology	−0.69 (0.40)	−0.71 (0.40)
Surgery	0.77 (0.58)	0.67 (0.58)
Pulmonology	0.02 (0.84)	0.10 (0.85)
Gastroenterology	−0.64 (0.60)	−0.75 (0.60)
Day admission	0.13 (0.46)	0.16 (0.46)
No admission/unknown	−2.73 (0.43)***	−2.71 (0.43)***
Other departments	0.36 (0.57)	0.39 (0.58)
Age patient		0.02 (0.01)
Sex patient		−0.28 (0.22)
**Random effects**		
ICC	33.31	33.38
Hospital level variance	1.64 (0.63)**	1.58 (0.61)**

*ICC* Intraclass Correlation Coefficient.

^a^Model 1 included successful hydration + admission department.

^b^Model 2 included model 1 + patients age and sex.

**P < 0.01, ***P < 0.001.

There was a negative association between a high eGFR (53–60) and hydration (Table 4), meaning that high-risk patients with a higher eGFR were less often hydrated compared to those with an eGFR value below 38 (ICC 24.00).

**Table 4:** Multi-level analysis of the association between eGFR value and hydration of high-risk patients (n = 561)

	**Model 1**^**a**^	**Model 2**^**b**^
	**Estimate (SE)**	**Estimate (SE)**
**Fixed effects**		
Successful hydration (constant)	1.39 (0.34)	1.42 (0.35)
eGFR value		
<38 ml/min/1.73 m^2^	*Reference*	*Reference*
38-44 ml/min/1.73 m^2^	0.38 (0.36)	0.34 (0.37)
45-52 ml/min/1.73 m^2^	−0.50 (0.31)	−0.51 (0.32)
53-60 ml/min/1.73 m^2^	−1.86 (0.31)***	−1.87 (0.31)***
Age patient	-	0.02 (0.01)
Sex patient	-	−0.23 (0.21)
**Random effects**		
ICC	24.00	23.77
Hospital level variance	1.04 (0.42)*	1.03 (0.42)*

*eGFR* estimated Glomerular Filtration Rate; *ICC* Intraclass Correlation Coefficient.

^a^Model 1 included successful hydration + eGFR value.

^b^Model 2 included model 1 + patients age and sex.

*P < 0.05, ***P < 0.001.

## Discussion

The eGFR, used for defining high-risk patients for CIN, was known in almost all patients (96%) undergoing contrast administration in Dutch hospitals. However, the subsequent step in the prevention of CIN was performed in fewer patients, as only on average 69% of the high-risk patients for CIN were hydrated before contrast administration. Over the course of the evaluation study in which the hospitals were expected to fully implement the Safety Program, there was no significant change in the rate of hydration of high-risk patients. A reasonable percentage of the total variance in hydration was caused by differences between individual hospitals. Admission department and eGFR value were statistically significantly associated with hydration, showing that those with a higher eGFR were less often hydrated compared to those with a lower eGFR value, and those not (or unknown) admitted were on average less often hydrated compared to the internal medicine department.

This is the first study investigating associations between hospital-related and admission-related factors and hydration of high-risk patients for CIN. If a patient was not (or unknown) admitted, only one out of three high-risk patients was hydrated. This low hydration rate seems to be explained mainly by the lack of time when a patient is visiting the hospital for one day, which was mostly an outpatient clinic visit. If patients had to undergo contrast administration acutely, there is insufficient time for hydration. The Safety Program module advises to hydrate these patients with a shortened hydration scheme [18]. However, in this case we would have also expected an association between acute admission and hydration. Patients at high risk were more often acutely admitted, but there was no difference in hydration between those acutely admitted and those not acutely admitted. Furthermore, non-statistically significant those admitted for one day were often hydrated. This seems to be the result of the Safety Program, as outpatients at high-risk for CIN scheduled for contrast administration would probably be hydrated at the day admission department. As most day admissions are planned, the eGFR measurement and hydration can also be scheduled. Another possible explanation for the differences between departments in general, was the difference between hospital protocols concerning the categorization of high-risk groups for CIN. For example, different risk factors were included in a hospital checklist and the definition of an abnormal eGFR can differ between the hospitals. If a higher cut-off value for eGFR was used by hospitals, this may explain why high-risk patients with a lower eGFR were more often hydrated compared to high-risk patients in the highest eGFR category. In addition, hydration of patients with a very low eGFR value can probably be seen as more urgent than hydration of high-risk patients with an eGFR value just below the cut-off point. However, the usefulness of hydrating lower risk patients for CIN (based on the absence of diabetes mellitus, age > 65 year, sCr >1.4 mg/dl) to prevent the incidence of CIN was suggested in a non-randomized controlled trial [25].

### Strengths and limitations

A strength of this study was the large representative sample of hospitals, which enables generalizability of the results to the national hospital population. Because of the large sample of patient records a distinction could be made between different types of departments and hospitals. However, this separation was only possible in the multi-level analysis with all measurements together, because otherwise the numbers within one measurement would be too small to perform separate analyses.

As this study was a patient record review study, the data was based on the information registered in the patient records. A limitation of this study design was that we only had information about the registered eGFR value and risk factors mentioned in the record and we did not know whether the hospital actually recognized high-risk patients. Possibly, not all risk factors included in the guideline are known when a physician applied contrast administration. Furthermore, if hydration was not registered in the patient record, we cannot be sure that the hydration was executed but incorrectly registered in the report. However, if hydration was not registered, this information could similarly not be found by other healthcare providers, which may have a negative effect on patient safety caused by an increased risk of under- or overtreatment. Information about hydration was missing in 46 records of the 627 high-risk patients (7%) and these patients had to be excluded from further analysis. If it was unknown whether a patient was admitted to the hospital (department category “no admission/unknown”), it seems reasonable that more information was lacking in the report. Finally, we only have information about the identification and hydration of high-risk patients and do not know to what extent CIN was truly prevented in these patients.

### Future research and practice

The hydration of high-risk patients is part of the module supporting the prevention of CIN and is based on national guidelines with preventing methods for contrast medium administration [17,18]. During the Safety Program discussion arose about the necessity of hydration within high-risk patients with specific contra-indications, because it was argumented that pre-hydration of these patients may be dangerous. Furthermore, a recent discussion paper questioned the evidence for CIN caused by contrast administration within patients with an eGFR > 30 ml/min [26]. The authors stated that prevention of CIN by hydration increases the health care costs and is also a burden for the patients because of longer hospital stay. However, the effect of hydration in reducing CIN in high-risk patients was shown in several studies. Although most of these studies did not include a control group [10,12-14], two RCT’s were performed with a placebo group and showed a statistically significant effect on reducing CIN [11,15]. No information was available about the costs of hydration and future studies should therefore focus more on the costeffectiveness of hydration of high-risk patients.

In practice, there should be more uniformity in the definition of high-risk patients. Our study showed a wide variety between the participating hospitals in using the national guidelines. There might be arguments to deviate from the protocol if hydration is not desirable for some reason, but it is important to argument and report these exceptions. The importance of assessing the eGFR and registration of the eGFR and subsequent interventions in the record to prevent CIN should be emphasized.

## Conclusions

There is clearly attention for the identification of high-risk patients for CIN, but the adherence to the subsequent step in the prevention of CIN was less often performed, as only two thirds of the high-risk patients were hydrated before contrast administration. Special attention is needed for high-risk patients with a relatively higher eGFR and not (or unknown) admitted high-risk patients, as hydration was statistically significantly less executed in these patients. Deviation of the guidelines may be caused by lack of time and possibly by the use of different criteria for high risk patient by the hospitals. Registration of identification and hydration of high-risk patients is important to give insight in the preventive methods, the validity of possible deliberate deviations and to facilitate the feedback of results to health care providers.

## Abbreviations

CIN: Contrast-induced nephropathy; eGFR: estimated glomerular filtration rate; sCr: serum creatinine.

## Competing interests

The authors declare that they have no competing interests.

## Authors’ contributions

JS performed statistical analyses, interpreted the analytical results and wrote the manuscript; CB designed the study, organized the data collection and made critical revision to the manuscript; ML participated in the data analysis and interpretation; PS performed statistical analyses and made critical revision to the manuscript; CW designed the study design and edited the manuscript. All authors approved the final version of the manuscript.

## Pre-publication history

The pre-publication history for this paper can be accessed here:

http://www.biomedcentral.com/1471-2369/15/2/prepub
